# The budget impact of alteplase in the treatment of acute ischemic stroke in Egypt

**DOI:** 10.3389/fneur.2023.1220615

**Published:** 2023-11-08

**Authors:** Hany Aref, Nevine El Nahas, Gihan Hamdy Elsisi, Hossam Shokri, Tamer Roushdy

**Affiliations:** ^1^Department of Neurology, Faculty of Medicine, Ain Shams University, Cairo, Egypt; ^2^HTA Office, LLC, Cairo, Egypt; ^3^Department of Economics, American University in Cairo, Cairo, Egypt

**Keywords:** stroke, rt-PA, thrombolysis, Egypt, acute ischemic stroke

## Abstract

**Introduction:**

Stroke is the second leading cause of mortality worldwide. Five percent of all the disability-adjusted life years (DALYs) lost around the world are attributed to stroke. This study aimed to assess the economic burden of acute ischemic stroke (AIS) in Egypt and reveal the benefits of alteplase treatment by measuring the resource use and costs associated with this treatment compared to the standard of care and extrapolate the overall budget impact of alteplase to the local Egyptian setting over a 5-year time horizon from a societal perspective.

**Methods:**

A budget impact model was developed to estimate the impact of adding alteplase to the current treatment of AIS patients within the Egyptian healthcare setting. The efficacy data for both arms of the model were sourced from a systematic review of the literature. Resource use and cost data were sourced from a retrospective study. Proportions of patients potentially eligible for treatment and the treatment time distributions were estimated from an analysis of the results of this retrospective data collection. A univariate sensitivity analysis was conducted to assess the robustness of the model results. The input parameters varied between plausible extremes based on a review of available evidence.

**Results:**

The total annual costs with alteplase treatment [i.e., drug, symptomatic intracerebral hemorrhage (ICH) management, acute hospitalization, and post-hospitalization costs] for the targeted patients from a societal perspective were estimated to be less than the total annual costs without alteplase. This resulted in savings of approximately EGP 37.2 million ($ 1.2 million), EGP 14.2 million ($ 458.06), EGP −33.0 million ($ −1.06 million), EGP −54.0 million ($ −1.74 million), and EGP −89.8 million ($ −2.89 million) for each of the 5 years, respectively. In year 1, more than 2,787 patients (+30.1%) achieved an excellent outcome and <1,204 patients (−22.3%) had a poor outcome when treated with alteplase. The savings in acute hospitalization and post-hospitalization costs offset the increase in drug and ICH management costs in the alteplase group compared to treatment without alteplase. The total cumulative cost savings for alteplase in AIS patients were estimated at EGP −228,146,871 ($ −7,359,576) over 5 years.

**Conclusion:**

The budget impact model estimates suggest that from a societal perspective, alteplase is likely to be a cost-saving option for the treatment of AIS in Egypt due to the treatment benefits, resulting in savings in acute hospitalization and annual post-hospitalization costs.

## Introduction

Non-communicable diseases (NCDs) are responsible for 41 million deaths each year, which equates to 71% of deaths globally (1). Approximately 15 million of these deaths were due to a stroke or ischemic heart disease (2). In 2016, stroke remained the second top cause of mortality worldwide (3). In the same year, it was estimated that beginning from the age of 25 years, the risk of stroke over a lifetime was 25% for the global population (4). Subsequently, strokes alone account for ~4% of health expenditure in Western countries mainly due to costs incurred during patients' hospitalization after an AIS, which corresponds to 70% of the total cost of strokes in the following year (2). In addition, 5% of all the DALYs lost around the world are attributed to stroke (5). Furthermore, more than 7% of stroke-related mortality and more than 80% of DALY losses occur in low- and middle-income countries (6).

According to the World Stroke Organization, the global cost of stroke exceeds 890 billion dollars, and this burden is suspected to keep on the rise with the increase in the life expectancy of patients (7).

In Egypt, the average incidence rate is 202 new incidents of stroke per 100,000 life years, leading to an average prevalence of 613 stroke patients in every 100,000 life years in the population (8). Out of the different types, ischemic strokes are the most common, accounting for ~90% of all strokes (9). With such a disease burden, stroke treatment in Egypt faces many challenges. Some of those include delays in AIS treatment in hospitals and suboptimal public awareness about stroke symptoms. Nevertheless, the most impactful factor is the low rate of thrombolysis in stroke management as it is not fully adopted by most hospitals (10).

Thrombolytic drugs are the main treatment option for AIS. They benefit patients by addressing the decreased blood flow in the brain due to thrombus (blood clot) formation. Thrombolytic drugs trigger plasminogen to form plasmin (11), an enzyme that breaks down fibrin and thus dissolves the thrombi. Alteplase (Actilyse^®^, Boehringer Ingelheim) is a second-generation, recombinant tissue-type plasminogen activator (rt-PA) thrombolytic drug. It is synthesized using recombinant DNA technologies and is approved by the Food and Drug Administration Agency (FDA) for the treatment of AIS, acute myocardial infarctions, blocked catheters, and pulmonary embolisms (12).

In a double-blind randomized clinical trial that enrolled 821 patients, patients were divided into two groups in a 1:1 ratio. One group was administered 0.9 mg/kg alteplase through an intravenous injection, while the other group was administered placebo (13). The study aimed to assess the efficacy and safety of alteplase if given 3–4.5 h after the onset of stroke symptoms for the treatment of AIS, as up to this point, its efficacy and safety were not sufficiently investigated (13). The study concluded that when comparing alteplase with placebo taken 3–4.5 h after the onset of a stroke, alteplase was found to improve outcomes, with no significant difference in mortality rates (13). A single-arm, open-label clinical trial was conducted in 22 centers in Japan, which enrolled 100 patients, to investigate the efficacy and safety of 0.6 mg/kg alteplase as compared to the recommended dose of 0.9 mg/kg (14). The study found that alteplase at 0.6 mg/kg dose had comparable efficacy and safety profiles to the previously investigated dose of 0.9 mg/kg (14).

Finally, a meta-analysis combining 8 clinical trials, pooling a total of 2,775 participants, evaluated alteplase as a treatment for AIS. The endpoints considered included disability at 90 days, rates of parenchymal hemorrhage cases, and mortality rates (15). It was concluded that patients benefited from alteplase if given within a 4.5-h period after stroke symptoms onset (15). However, if the 4.5-h period was exceeded, the risk–benefit ratio of administering alteplase might be tipped toward risks rather than benefits (15).

Unlike other thrombolytic drugs such as streptokinase and urokinase, alteplase is not associated with increased morbidity or mortality at 3 months; nevertheless, different studies presented a favorable outcome at 90 days in cases of acute ischemic stroke who were treated by alteplase. Despite the current availability of tenecteplase in some countries and its usage under trials in acute ischemic stroke, alteplase is still the only gold-approved drug in AIS, whatever its etiology and whatever the caliber of the occluded vessel (16, 17).

Given the lack of data, this study assessed the economic burden of AIS in Egypt and the clinical benefit associated with the use of alteplase by measuring the resource use and costs associated with the management of stroke and extrapolating the overall budget impact of alteplase use over the local Egyptian setting over a 5-year time horizon from a societal perspective with data sources obtained from the safe implementation of treatments in the stroke database of Ain Shams University hospitals stroke unit with a targeted question whether alteplase use despite its initial cost is cost-effective on the long term in cases receiving it compared to historical controls who did not receive it.

Being an approved drug in AIS, it was not ethically applicable to withhold its administration to cases eligible to receive it so the cost-effectiveness of the current study was done in comparison to historical controls which might be considered a limitation in the study; yet, it is not possible to overcome for ethical and medicolegal aspects. Another limitation is the availability of thrombolytics and the capability of patients to reach stroke service providers at a suitable time to receive alteplase therapy; yet, this could be handled through advocacy and awareness campaigns by properly addressing the values of receiving alteplase over the misfortune of losing time to receive it.

## Methodology

### Retrospective data collection

In order to estimate the proportion of patients potentially eligible for thrombolysis based on the ability to treat within 4.5 h of symptom onset, and the distribution of treatment times for those who do receive treatment, an analysis of a retrospective data collection was conducted, as it was deemed feasible within the context of the available budget and timeframe in comparison to alternatives including an observational prospective cohort study or an interventional study. At Ain Shams University (ASU) Hospital, thrombolysis was gradually rolled out during the year 2013, and data were available from the following year in 2014. An extensive informatics system was in place at ASU Hospital for several years prior to thrombolysis implementation in 2013. Most of the inputs required for the data analysis were routinely collected as ASU Hospital was the first hospital in Egypt to join the safe implementation of treatments in stroke (SITS) database, which is an international database used by many countries worldwide with properly designed input protocols for different kinds of stroke and different management modalities including thrombolysis. Continuous consistency checking is being done by the SITS international headquarters on a monthly basis with awards presented to hospitals with complete data; this was well-recognized by the SITS international in the case of ASU Hospital and was readily available in the database, particularly, the time since onset of symptoms at presentation [typically expressed as onset-to-door (OTD) time] and the time between presentation and administration of treatment [typically expressed as the door-to-needle (DTN) time], the presence of the latter indicating administration of rt-PA treatment (and conversely, the lack of which indicating no rt-PA treatment), and the sum of both giving a reliable measure of the total time between onset of stroke and administration of rt-PA [typically expressed as the onset-to-needle (OTN) time]. The eligibility of rt-PA cases was based on the criteria of the European Cooperative Acute Stroke Study (ECASS and ECASS II) (18, 19).

Prior to enrolment into the SITS patients or their caregivers, they sign an informed medical consent involving the possibility of using the medical records in research yet with full confidentiality regarding the patient's personal information. Prior to retrieval of data from the database and analysis, the study received ethical approval from the IRB of the faculty of medicine, Ain Shams University under the code FMASU R20/2023; the study was conducted over a period covering the previous 5 years from the beginning of the study. Based on the patient databases available, this approach provided a sample size of several hundred patients and offered relatively robust data in a very short time. Preliminary feasibility discussions were conducted, and all the necessary inputs were collected over the study period.

Patients seen at ASU Hospital were assumed to be representative of the rest of the patient population across the country as ASU Hospital is a major tertiary referral hospital in the east of Cairo and is considered one of the largest medical institutes in the region that serves as a large catchment area including urban and rural areas with ~775,000 medical visits annually and 5,000 physicians in different fields of medicine and with a well-equipped and established stroke unit since the early of the 21st century. The main outcomes of this study were the resource use and costs associated with managing AIS during the acute phase and, where available, from the time of discharge (or transfer to a long-term care facility) and up to 1 year after that. The level of resource use was considered not to depend on whether the patient received rt-PA or not but instead on how severe their long-term disability was, captured by proxy with the modified Rankin Scale (mRS) score at 90 days, a measure of the patient's disability on a scale from 0 (no disability at all) to 6 (death). All cost estimates were stratified according to this disability level for all individuals. The percentage of patients using a resource and the annual frequency of that resource's use were based on local clinical practice and validated by local experts.

The 90-day mRS ratings were available for 70–80% of admitted patients, which represented a final sample of 378 patients (in both treatment arms). These data were only used to stratify the resource use associated with AIS treatment.

### Data validation

Resource use and cost data were closely monitored and regularly audited within ASU Hospital as they formed the basis of the billing and accounting systems. Data collection regarding other inputs was also done at the highest level of quality in accordance with good research practice. Final consistency checks were implemented before the analyses took place. We also followed the good practice of budget impact analysis report that was led by the International Society of Health Economics and Outcomes Research (ISPOR).

### Budget impact model

**Figure 1:**
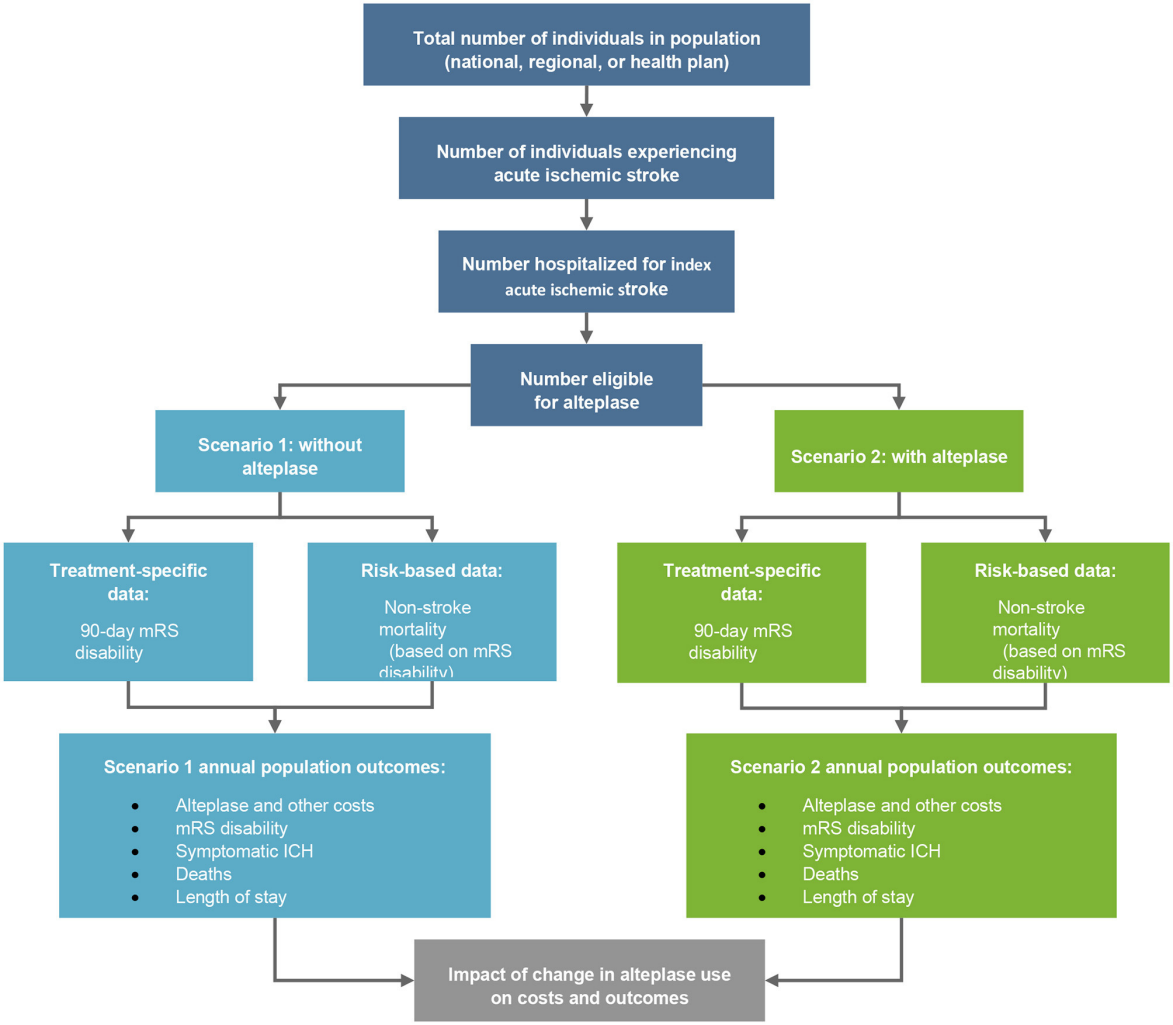
Budget impact model of alteplase. mRS, modified Rankin Scale; ICH, intracranial hemorrhage.

**Figure 2:**
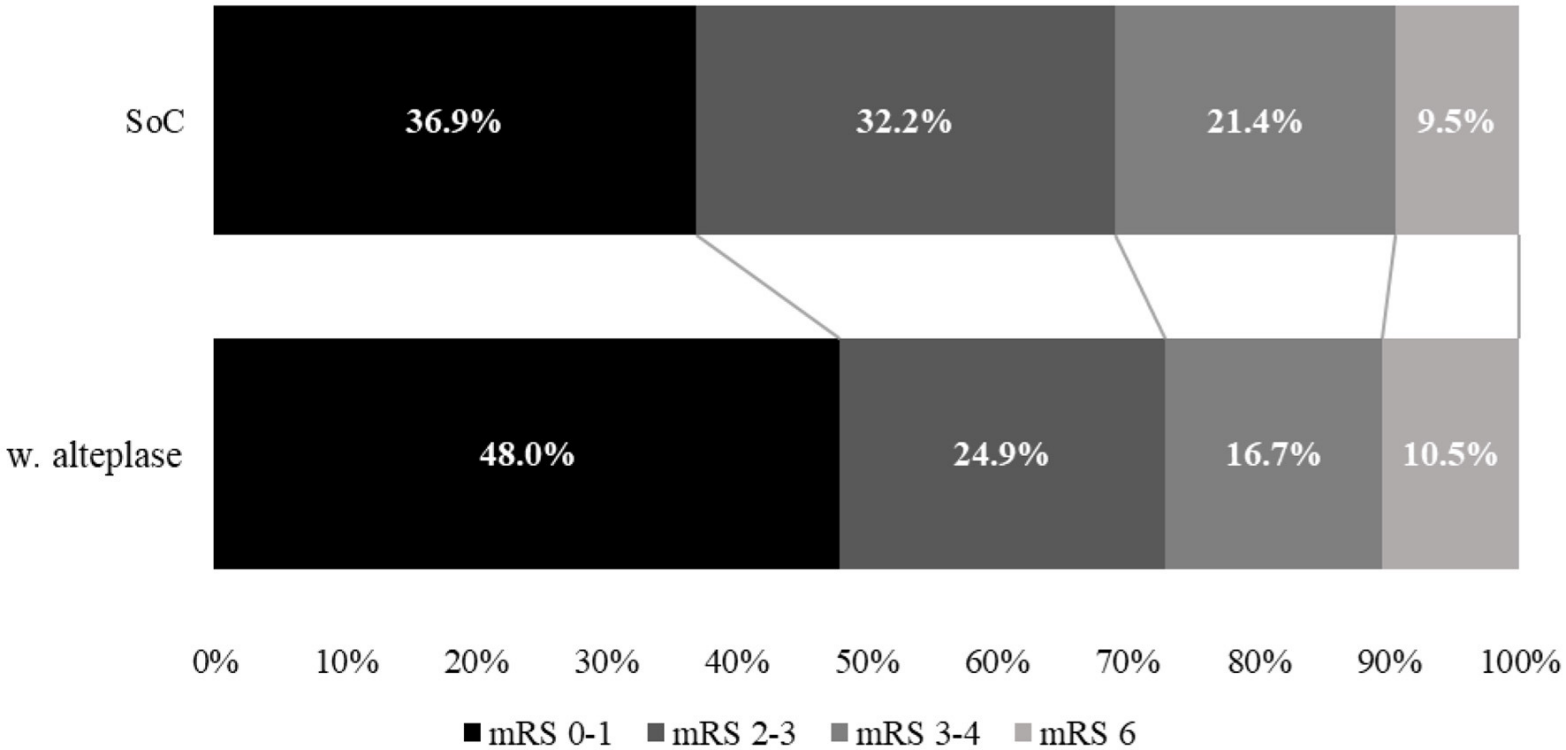
Comparison of mRS disability at 90 days between treatment with standard of care (SoC) and alteplase for year 1.

A budget impact economic model was developed using Microsoft Excel for Windows (Microsoft Office 365). It was a static decision-tree model, built from a societal perspective and the chosen time horizon was 5 years (Figure 1). The results from a systematic literature review provided efficacy and safety data for both arms. A retrospective data collection conducted at ASU Hospital was used to measure the resource use, costs, OTD, and DTN times associated with the management of AIS patients (Figure 2).

This study modeled a cohort of AIS patients eligible for rt-PA treatment (i.e., within 4.5 h from the onset of symptoms) through two possible arms. In the base case considered as the current scenario, patients were assumed to be treated without thrombolysis, whereas in a revised scenario, patients received alteplase. The OTN times were collected and grouped in intervals of 0–1.5 h, 1.5–3 h, and 3–4.5 h.

Clinical outcomes and costs by OTN time interval were then compared between the two scenarios to estimate the monetary impact of adding alteplase to the current treatment of AIS patients within the Egyptian healthcare setting. In the second phase, the impact of modifying the DTN time on the time distribution of the patients and their clinical outcomes and costs was investigated.

In the model, the current and revised model scenarios accounted for direct medical costs associated with the treatment of AIS patients within the Egyptian healthcare setting. A micro-costing approach was adopted for the costing analysis. All unit costs assigned in the model were obtained from public institutions and health insurance pricelists through access to CAPMAS and contacting different health insurance companies. Average annual costs after discharge (post-hospitalization) were also added considering a societal perspective. Costs were calculated in Egyptian pound (EGP).

To ensure that the simulation in the model was aligned with the current local practice for the treatment of these patients within the Egyptian healthcare setting, an expert panel including local neurology experts was consulted through virtual interviews to validate the model assumptions including the costs and length of stay within the different compartments of the hospital and whether stroke units, intensive care units, and ordinary beds are based on the medical condition of the patients and any associated possible complications as well as the ordered work up and investigations. The panel's feedback was analyzed, ensuring a consensus was reached between the experts.

The inputs and findings for this study and its model were checked for internal and external validity. The estimates for OTN times were compared with other studies from countries close to Egypt within the MENA region to ensure external validity. As for the patient journey and stages of treatment, those were validated by an expert panel to match Egyptian clinical practice. The results were also tested for robustness through a deterministic sensitivity analysis.

## Results

Outcome data for a total of 2,630 AIS cases (in 5 years) admitted between January 2016 and December 2020 were extracted from a retrospective database analysis of digital patient records in the neurology department of ASU Hospital. The average annual number of AIS cases admitted within the treatment window was estimated at 658, from which 526 were eligible for injection each year and 378 were followed for at least 90 days. Their baseline characteristics are shown in Table 1. Thrombolysis has been shown to reduce the 90-day disability of a patient as measured on the mRS (15).

**Table 1:** Baseline characteristics of AIS patients in ASU Hospital.

**Parameter**	**Percentage**
Male (sex)	60.64%
Age (>65 years)	39.35%
**NIHSS score at admission**	
3–7	12.75%
8–14	42.83%
15–21	42.84%
> 21	1.58%
**Smoking**	
Current smoking	11.4%
Ex-smokers	3.04%
Hypertension	59.12%
Atrial fibrillation	17.87%
History of myocardial infarction	2.66%
Congestive heart failure	3.23%
Diabetes mellitus	37.45%
**Prior stroke**	
Stroke earlier than 3 months	9.69%
Stroke within 3 months	1.14%

### Budget impact model

#### Target population

The budget impact model was based on the total number of AIS cases in Egypt, estimated to be 25,140 lives (8, 9), as shown in Table 2. A total population of 102,334,404 was sourced from UN projections for mid-year 2021. As published in the literature, the average crude incidence rate of stroke in Egypt, weighted by sample population size, was 202 out of 100,000 (8). The estimated percentage of AIS was 60–90.1% of total stroke patients, making them the most common type of stroke reported (9). Therefore, a 90% incidence of AIS was modeled based on the published literature and feedback from the expert panel, resulting in an incidence rate of 181.8 out of 100,000 for AIS specifically. The percentage of patients who were actually hospitalized for an AIS was 79.4% (20). A thrombolysis rate of 17.0% was extracted from the local retrospective data collected from ASU Hospital. Patients were considered eligible for treatment if they arrived, were assessed, and deemed suitable for treatment within 4.5 h of symptom onset.

**Table 2:** Epidemiology and target population for acute ischemic stroke (AIS) in Egypt.

	**Rate**	**Population**	**References**
Total population (*n*)		102,334,404	UN projections
Annual incidence of AIS (n; per 100,000)	181.8	186,044	(7, 8)
Percentage hospitalized for an AIS	79.4%	147,739	(15)
Percentage arriving in time and eligible for thrombolysis	17.0%	25,140	ASU Hospital

#### Treatment scenarios

The current scenario (no thrombolysis) assumed that patients received the standard of care (SoC) regimen (assuming no cost included in the model). AIS patients who were eligible to receive rt-PA treatment were administered two intravenous injections of alteplase 50 mg infusion plus SoC (only the cost of alteplase was included). The dosage was as indicated in the drug leaflets and validated by the expert panel. It is assumed that 100% of patients eligible for alteplase treatment did receive it in the revised scenario, as was observed in practice. The figures for both scenarios are shown in Table 3.

**Table 3:** Treatment scenarios.

**Current scenario**	**Year 1**	**Year 2**	**Year 3**	**Year 4**	**Year 5**
SoC	100%	100%	100%	100%	100%
With alteplase	0%	0%	0%	0%	0%
**Revised scenario**	**Year 1**	**Year 2**	**Year 3**	**Year 4**	**Year 5**
SoC	0%	0%	0%	0%	0%
With alteplase	100%	100%	100%	100%	100%

SoC, standard of care.

#### Clinical parameters

The clinical parameters in the three time intervals, within 4.5 h from onset of symptoms and stratified by mRS score at 90 days for both scenarios, were extracted from a meta-analysis which included several major clinical trials reported in the literature (ECASS I, II, and III, NINDS 1 and 2, ATLANTIS 1 and 2, and EPITHET) (15) and was validated for local use by the expert panel (Table 4).

**Table 4:** 90-day mRS efficacy outcomes per time interval for both scenarios (14, 17).

**Standard of care**	**0–1.5 h**	**1.5–3 h**	**3–4.5 h**
mRS 0–1	31.50%	35.20%	40.88%
mRS 2–3	37.71%	32.33%	30.79%
mRS 4–5	15.08%	23.86%	18.74%
mRS 6	15.72%	8.62%	9.60%
**With alteplase**			
mRS 0–1	53.97%	47.11%	48.09%
mRS 2–3	23.81%	24.89%	25.14%
mRS 4–5	9.52%	18.37%	15.30%
mRS 6	12.70%	9.63%	11.47%

The model also accounted for the increased incidence of ICH associated with alteplase. These data were captured from the meta-analysis mentioned previously (15) and were also validated by the expert panel.

The annual risk of non-stroke death was derived from the crude death rate per 1,000 for Egypt (21). The relative risk for death was adjusted by the 90-day mRS disability level (22).

#### Treatment times

The OTN distributions from ASU Hospital between 2016 and 2020 provided the time to treatment inputs for the three time intervals (0–1.5 h, 1.5–3 h, and 3–4.5 h) over the 5-year time horizon (Table 5).

**Table 5:** Distribution of onset-to-needle time per year.

**Onset-to-needle time**	**2016**	**2017**	**2018**	**2019**	**2020**
0–1.5 h from onset	7.5%	5.3%	6.3%	17.1%	16.7%
1.5–3 h from onset	58.2%	43.4%	57.1%	52.3%	83.3%
3–4.5 h from onset	34.3%	51.3%	36.6%	30.6%	0.0%

Admittedly, 2020 also corresponds to the peak of the COVID-19 pandemic, which clearly impacted the time distribution for that year. Overall, fewer patients sought treatment, and when they did, they were able to access the hospital much faster, thanks to the reduced traffic. Additionally, fewer caregivers were at their usual workplaces, thereby likely nearer to their patients at the time of stroke onset. These factors could largely explain how all patients were treated within 3 h.

#### Costs and resource use

Direct medical costs for acute hospitalization costs included rt-PA drug costs, hospitalization (ICU and non-ICU hospital stay and intermediate stroke unit), surgery procedures, general imaging procedures, laboratory investigations, physical examinations, concomitant medications, physiotherapy sessions, assisted feeding, and ICH management costs. Annual post-hospitalization costs involved subsequent hospitalizations (including discharge to a second hospital), speech and language therapy, physiotherapy and neurologist follow-up visits, and also home care from a societal perspective. All resource use and costs were then stratified by mRS score at 90 days to reflect the intensity of care required by patients in each category, during the acute (Table 6) and post-hospitalization (Table 7) phases.

**Table 6:** Acute hospitalization resource use and unit costs for AIS patients by 90-day mRS.

**Acute hospitalization**	**mRS 0–1**	**mRS 2–3**	**mRS 4–5**	**mRS 6 (death)**
**Non-ICU hospital day**				
Used by (% patients)	100%	50%	35%	35%
Cost per day	EGP 550 ($ 17.7)			
Length of stay (days)	4	2	1	1
**ICU hospital day**				
Used by	0%	30%	70%	70%
Cost per day	EGP 2,250 ($ 72.58)			
Length of stay (days)	7	15	30	30
**Intermediate care stroke unit**				
Used by	0%	50%	65%	65%
Cost per day	EGP 1,200 ($ 38.71)			
Length of stay (days)	–	3	4	4
**Surgical procedures**				
Used by	0%	30%	70%	70%
Cost per procedure	–	EGP 10,000 ($ 322.58)		
Number of procedures	–	1	1	1
**General imaging procedures**				
Used by	100%	100%	100%	100%
Cost per procedure	EGP 2,750 ($ 88.71)			
Number of procedures	1	1	1	1
**Concomitant medication**				
Used by	100%	100%	100%	100%
Cost per dose	EGP 600 ($ 19.35)			
Number of doses	5			
**Assisted feeding**				
Used by	0%	0%	100%	100%
Costs per feeding	–		EGP 200 ($ 6.45)	
Number of feedings	–		6/day ^*^ 5 days = 30	
**Laboratory investigations (routine lab tests)**				
Used by	100%	100%	100%	100%
Costs per test	EGP 800 ($ 25.81)			
Number of tests	1	1	1	1
**Physical examinations**				
Used by	100%	100%	100%	100%
Costs per examination	EGP 100 ($ 3.23) (outpatient visit)			
Number of examinations	4	5	5	5
**Physiotherapy sessions**				
Used by	0%	80%	100%	100%
Costs per session	EGP 200 ($ 6.45)			
Number of sessions	–	4	4	4
**Speech and language therapy**				
Used by	0.0%	50.0%	65.0%	65.0%
Cost per procedure	EGP 250 ($ 8.06)			
Number of procedures	–	15	36	36

**Table 7:** Post-hospitalization resource use and unit costs for AIS patients by 90-day mRS.

**Post-hospitalization**	**mRS 0–1**	**mRS 2–3**	**mRS 4–5**	**mRS 6 (death)**
**Discharge to the second hospital**				
Used by	0%	5%	10%	–
Cost per day	EGP 550 ($ 17.74)			–
Length of stay	–	7	15	–
**Home care**				
Used by	0%	65%	65%	–
Costs per day	–	EGP 750 ($ 24.19)	EGP 1,500 ($ 48.39)	–
Number of sessions	–	25	30	–
**Speech and language therapy**				
Used by	0%	50%	65%	–
Costs per session	EGP 250 ($ 8.06)			–
Number of sessions	–	15	3^*^52 = 156/year	–
**Physiotherapy**				
Used by	20%	100%	100%	–
Cost per session	EGP 200 ($ 6.45)			–
Number of sessions per year	10	20	208	–
**Neurologist follow-up visits**				
Used by	100%	100%	100%	–
Cost per visit	EGP 200 ($ 6.45)			–
Number of visits per year	2	3	5	–
**Other rehabilitation resources**				
Used by	0%	20%	40%	–
Cost per procedure	EGP 400 ($ 12.90)			–
Sessions per year	–	12	24	–
**General practitioner follow-up visits**				
Used by	0%	70%	100%	–
Cost per visit	EGP 150 ($ 4.84)			–
Visits per year	–	4.00	10.00	–

### Budget impact analysis results

As expected from the meta-analysis used as the source for clinical inputs, improved 90-day disability outcomes when alteplase is added to SoC. For example, in year 1, more than 2,787 patients (+30.1%) were projected to experience an excellent outcome (mRS 0–1 at 90 days), and >1,204 patients (−22.3%) were projected to experience a poor outcome (mRS 4–5 at 90 days). While disability decreases, fewer resources are consumed, and in particular, patients spent < 101,933 days in hospital beds (−8.2%) over 5 years, freeing up valuable resources for the healthcare system.

The model's economic outputs were consolidated as drug costs, acute hospitalization costs, ICH management costs, and post-hospitalization costs, all expressed for the total population of Egypt.

From a societal perspective, the total annual costs in the revised scenario (alteplase arm) were estimated to be less than the total annual costs in the current scenario (SoC without alteplase), leading to potential cost savings of approximately EGP −37.2 million ($-1.2 million), EGP −14.2 million ($-458.06), EGP −33.0 million ($-1.06 million), EGP −54.0 million ($-1.74 million), and EGP −89.8 million ($-2.89 million) for each of the 5 years, respectively (Table 8). Most of the cost savings were achieved in the post-hospitalization phase, with a reduction of EGP −813,488,333 ($-26.241.559) over 5 years. Together, the savings in acute hospitalization and post-hospitalization costs after introducing alteplase fully offset the increase in drug and ICH management costs, observed over SoC without alteplase. The total cumulative savings for alteplase in AIS patients were estimated at EGP −228,146,871 ($-7.359.576) over 5 years.

**Table 8:** Budget impact of alteplase compared to standard of care (SoC) for acute ischemic stroke patients in Egypt from a societal perspective (in EGP and its transaction to $ US).

	**Year 1**	**Year 2**	**Year 3**	**Year 4**	**Year 5**
**Total costs**					
Standard of care	EGP 1,617,044,503 ($ 52,162,725,90)	EGP 1,584,895,529 ($ 51,125,662,22)	EGP 1,614,659,099 ($ 52,085,777,39)	EGP 1,604,901,302 ($ 51,771,009,74)	EGP 1,672,120,878 ($ 53,939,383,16)
With alteplase	EGP 1,579,841,255 ($ 50,962,621,13)	EGP 1,570,693,939 ($ 50,667,546,42)	EGP 1,581,702,682 ($ 51,022,667,16)	EGP 1,550,928,077 ($ 50,029,037,97)	EGP 1,582,308,488 ($ 51,042,209,29)
Net budget impact	EGP −37,203,248 ($ −1,200,104)	EGP −14,201,590 ($ −458,115)	EGP −32,956,417 ($ −1,063,110)	EGP −53,973,225 ($ −1,741,071)	EGP −89,812,390 ($ −2,897,173)
**Drug costs**					
Standard of care	0	0	0	0	0
With alteplase	EGP 214,943,900 ($ 6,933,674)	EGP 214,943,900 ($ 6,933,674)	EGP 214,943,900 ($ 6,933,674)	EGP 214,943,900 ($ 6,933,674)	EGP 214,943,900 ($ 6,933,674)
Net budget impact	EGP 214,943,900 ($ 6,933,674)	EGP 214,943,900 ($ 6,933,674)	EGP 214,943,900 ($ 6,933,674)	EGP 214,943,900 ($ 6,933,674)	EGP 214,943,900 ($ 6,933,674)
**ICH costs**					
Standard of care	EGP 299,925 ($ 9,675)	EGP 372,482 ($ 12,015)	EGP 308,887 ($ 9,964)	EGP 291,464 ($ 9,402)	EGP 157,022 ($ 5,065)
With alteplase	EGP 1,494,110 ($ 48,197)	EGP 1,627,775 ($ 52,508)	EGP 1,506,316 ($ 48,590)	EGP 1,521,674 ($ 49,086)	EGP 1,253,232 ($ 40,426)
Net budget impact	EGP 1,194,186 ($ 38,522)	EGP 1,255,294 ($ 40,493)	EGP 1,197,428 ($ 38,626)	EGP 1,230,210 ($ 39,684)	EGP 1,096,210 ($ 35,361)
**Acute hospitalization costs**					
Standard of care	EGP 888,271,441 ($ 28,653,917)	EGP 875,351,651 ($ 28,237,150)	EGP 886,611,061 ($ 28,600,356)	EGP 890,424,583 ($ 28,723,373)	EGP 914,052,806 ($ 29,485,574)
With alteplase	EGP 794,311,291 ($ 25,622,944)	EGP 793,176,914 ($ 25,586,352)	EGP 795,105,630 ($ 25,648,568)	EGP 785,078,143 ($ 25,325,101)	EGP 791,688,201 ($ 25,538,329)
Net budget impact	EGP −93,960,150 ($ −3,030,972)	EGP −82,174,738 ($ −2,650,798)	EGP −91,505,431 ($ −2,951,788)	EGP −105,346,440 ($ −3,398,272)	EGP −122,364,605 ($ −3,947,245)
**Annual post–hospitalization costs**					
Standard of care	EGP 728,473,137 ($ 23,499,133)	EGP 709,171,396 ($ 22,876,496)	EGP 727,739,150 ($ 23,475,456)	EGP 714,185,255 ($ 23,038,234)	EGP 757,911,050 ($ 24,448,743)
With alteplase	EGP 569,091,954 ($ 18,357,804)	EGP 560,945,350 ($ 18,095,011)	EGP 570,146,837 ($ 18,391,833)	EGP 549,384,361 ($ 17,722,076)	EGP 574,423,156 ($ 18,529,779)
Net budget impact	EGP −159,381,183 ($ −5,141,328)	EGP −148,226,046 ($ −4,781,485)	EGP −157,592,314 ($ −5,083,623)	EGP −164,800,894 ($ −5,316,157)	EGP −183,487,895 ($ −5,918,964)

### Sensitivity analysis

To test the robustness of the model and its sensitivity to variation in the parameters of the model, a deterministic sensitivity analysis was performed that modified one component at a time to assess its impact on the total cost. Within the sensitivity analysis, individual parameters were varied over a range from low to high by increasing and decreasing their values by 20%.

**Figure 3:**
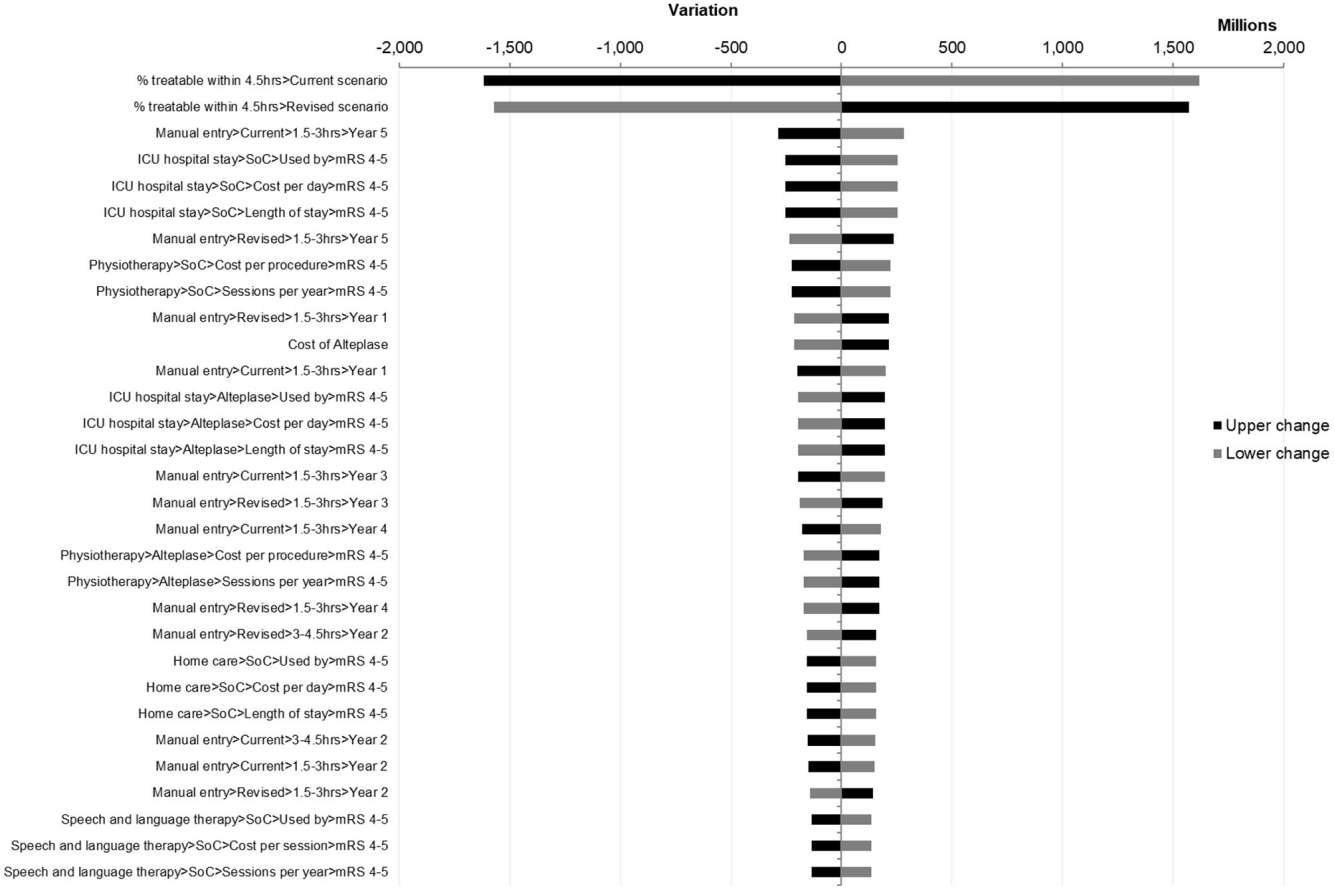
Deterministic sensitivity analysis tornado diagram.

The results of one-way sensitivity analyses were presented in a Tornado diagram, which showed the budget impact with low and high parameter estimates arranged from most impactful to least impactful (Figure 3).

The most impactful factor was found to be the proportion of patients treatable within 4.5 h, which is unlikely to vary significantly.

Parameters associated with ICU hospital stay (use rate, length of stay, and cost per day) were found to be impactful, with the lower bound resulting in the 5-year net budget impact becoming neutral at EGP 25,647,697 ($ 827,345), which only represents an increase of 0.32%. Additionally, it is unlikely for these parameters to vary significantly as they were based on actual hospital data and reviewed by an expert panel.

Finally, one more impactful factor would be the proportion of patients treated in the 1.5–3-h time interval in year 5. As the OTN distribution for this year differed significantly from the 4 years preceding it due to the impact of COVID-19, it may be worth noting that a distribution such as this, with the potential to be impactful on the result, is not typical or expected in normal conditions. No further parameters had the potential to alter the final conclusions of this budget impact analysis.

It is also worth noting that the parameters associated with home care caused significant variations of the net impact, from EGP −385,257,793 ($ −12,427,670) for the lower band to EGP −71,035,948 ($ −2,291,482) for the upper band, with a total amplitude of EGP 314,221,846 ($ 10,136,188). Meanwhile, in both cases, the model still showed cost savings at a sensitivity deviation of ±20%, demonstrating that a societal perspective is not necessary to achieve savings.

## Discussion

Thrombolysis with alteplase is already approved in Egypt for the management of AIS within 4.5 h of symptom onset. The aim was to assess the economic burden of acute ischemic stroke in Egypt and reveal the benefits of alteplase treatment by measuring the resource use and costs associated with this treatment compared to SoC and extrapolating the overall budget impact of alteplase to the local Egyptian setting over a 5-year time horizon from a societal perspective. Additionally, insight was sought on the impact of incremental reductions in mean OTN times.

In Egypt, alteplase is recommended for use within 4.5 h of symptom onset (22). This is similar to the recommended practice in countries such as the United States, Canada, South Africa, India, South Korea, China, Switzerland, the UK, and European Union guidelines (22). Ideally, 25% of all AIS patients should be eligible for treatment with thrombolytic drugs within 4.5 h of symptom onset. However, clinical practice does not reflect this. In the European Union, only 7.3% of the patients received thrombolysis, a percentage that is expected to be even less in middle- to low-income countries (22). However, as the AIS burden increases and the importance of thrombolysis is highlighted, many countries may consider working to decrease the OTN time to increase the proportion of patients potentially eligible for treatment by thrombolysis (since it must be administered within 4.5 h) and/or decreasing their average DTN time to ensure a greater proportion of those presenting in time can also be treated within 4.5 h. For instance, the European Stroke Organization has declared that it aims to double its thrombolysis rate to 15% by 2030 (22).

One of the most impactful factors in decreasing the time between the onset of the symptoms and the administration of alteplase is the DTN time (23). The American Heart Association/American Stroke Association started a project in 2010 with the aim of setting the DTN time at <1 h for half the patients administered (23). In 2014, the same objective was set for 75% of the patients. Finally, in 2016, the aim was to limit the DTN time to <45 min in half the patients. In this study, the onset-to-door times are within the margin of what was seen in other countries and the door-to-needle time could be reduced to 30 min for all patients. These goals demonstrated the room for improvement in AIS management not only in regard to medication but also in terms of improving the healthcare system services, which will subsequently lead not only to lives being saved but also to cost containment and resource use minimization. Not only is this cost reduction due to a decrease in direct medical cost but also the avoided complications and morbidity lead to an increase in the productivity of the patients and thus returns a benefit to the whole society. Furthermore, the improvement of the healthcare services to allow for the administration of alteplase in the recommended time will result in better outcomes and subsequently better health-related quality of life and patient satisfaction. A study published by the American Neurological Association in 2021 highlighted the benefits associated with the 30-min door-to-needle time standard for alteplase administration. A total of 752 AIS patients were included in a retrospective case–control study and were treated with intravenous alteplase in a large academic health system during 2015–2018 and compared their outcomes after treatment within 30, 45, and 60 min of arrival. They used an adjusted logistic and linear regression analysis to estimate the effects, after adjusting for baseline characteristics. The results of this study illustrated that there was a significant improvement in clinical efficiency of care outcomes as a result of early intravenous alteplase treatment within 30 min of hospital arrival. The lower DTN time categories were associated with improved clinical outcomes. One of the outcomes compared in this study was the length of stay (LoS). Patients who received treatment within 30 min of arrival had a statistically significant reduction in LoS in comparison to those patients who received the treatment within 31–180 min of arrival. The most obvious benefit associated with reduced length of stay is that it also reduces the risk of nosocomial infections and medication-related adverse events. Second, due to fewer days spent in the hospital, it reduces the burden on health insurance providers and out-of-pocket payments for the patient which in turn minimizes the societal cost (24).

The results of this study indicate that the treatment of AIS with alteplase during the 4.5-h period after stroke symptoms onset results in substantial cost savings over a 5-year time horizon. This is due to fewer acute hospitalizations, lower annual post-hospitalization costs, and fewer patients with disabilities from a societal perspective, which offset the increase in treatment cost. When examining the benefits of alteplase from a societal perspective, it is important to consider the indirect benefits associated with reduced days in the hospital. This will increase the quality of life for the patient which will in turn allow the patient to work and increase their level of wealth and contributions to society, while also freeing up beds and resources in a context of often saturated hospitals.

Previous studies were conducted in various settings to assess the budget impact of alteplase in the treatment of AIS. In Turkey, it was found that the use of alteplase would have a budget-saving effect on the Turkish healthcare system (25). Furthermore, the use of alteplase would result in a decrease in disability in the long term, thus generating further budget savings (25). In Spain, it was found that the extra cost of treatment would exceed the generated savings in the first 6 years; however, beyond that, the use of alteplase would have a long-term saving effect on the budget (26).

Other studies included cost-effectiveness analyses and were conducted in various countries including China, Denmark, the United Kingdom, and the United States (27–30). In Denmark, a study to assess the cost-effectiveness of thrombolysis accompanied by magnetic resonance imaging (MRI) vs. conservative treatment found that while the use of alteplase is not cost-effective in the short term, it is the dominant treatment in the long term (27). In addition, a study was conducted in the US to assess the use of rt-PA within 0–3 h after the onset of symptoms for AIS, from a third-party perspective, which found that treatment with rt-PA is cost-effective (28). Another study in China confirmed those conclusions and found that rt-PA treatment is highly cost-effective (21). Furthermore, in the UK, the health technology assessment of alteplase revealed that thrombolysis with alteplase is cost-effective and has an ICER of EUR 14,026 per QALY gained (29). A literature review of alteplase economic evaluations published from 1995 to 2016 included 16 studies that evaluated alteplase administrated at varying intervals after the onset of symptoms (21). The time intervals investigated were 0–3 h, 3–4.5 h, 0–4.5 h, and 0–6 h after the onset of the symptoms (30). The review concluded that alteplase is the dominant treatment compared to conventional treatment in the management of AIS (30).

## Limitations

The rates for events and complications for both scenarios were extracted from a meta-analysis but may differ from real-world data. To address this limitation, incidence and rates were validated by an expert panel to ensure fit for the Egyptian population. Another limitation of the analysis is that the study did not capture the impact of the Egyptian real-world circumstances on certain inputs. For instance, the public transportation infrastructure of Cairo and the dense traffic might further increase the time between the onset of symptoms and arrival at the hospital compared to other regions outside of Cairo.

The generalizability of these results across the Egyptian population may be limited by the fact that the clinical practice and availability of healthcare services might differ in governorates other than Cairo which, as the capital, accounts for around a fifth of the Egyptian population (24, 31). The prevalence of the disease and the quality of healthcare services may vary in rural areas further from the capital. To address this limitation, the findings could be limited to the population within Cairo, or the model could be adjusted to reflect the characteristics of other areas including prevalence data, patient journey, and time to treatment initiation.

Adequate thrombolytic treatment and specialized structures are not available everywhere in Egypt: Thrombolysis rates are locally comparable to established markets where stroke units already exist, particularly in Cairo (10–20% locally). However, coverage at the national level still needs to be improved. The resulting burden of loss of quality of life, disability, and ultimately premature death for the patients, and beyond for the society, could ideally be alleviated through two potential directions: first, by improving the general public's education in recognizing the symptoms of stroke (e.g., F.A.S.T. campaign in English-speaking countries such as the USA or the UK), and second, by promoting adequate patient management via dedicated stroke units and appropriate use of rt-PA.

## Conclusion

From a societal perspective, model results suggest that alteplase is likely to be a cost-saving option for the treatment of AIS in Egypt. These findings were driven by a reduction in patient disability and savings in acute hospitalization and annual post-hospitalization costs. Additionally, disability is likely to decrease, and patients will likely spend fewer days in the hospital and free up beds and other resources for the healthcare system. Results were found to be robust following a sensitivity analysis which varied input parameters by plausible extremes.

## Data availability statement

The original contributions presented in the study are included in the article/supplementary material, further inquiries can be directed to the corresponding author.

## Ethics statement

Ethical review and approval was not required for the study on human participants in accordance with the local legislation and institutional requirements. Written informed consent from the patients/participants or patients/participants' legal guardian/next of kin was not required to participate in this study in accordance with the national legislation and the institutional requirements.

## Author contributions

HA and NE: interpretation of data, editing, and revision of manuscript. GE: statistical analysis and writing manuscript. HS: recruitment, revision manuscript, and submission. TR: research idea and execution, recruitment, retrieving data, and writing manuscript. All authors contributed to the article and approved the submitted version.

## References

[1] WHO. Non Communicable Diseases. Who.int. (2021). Available online at: https://www.who.int/news-room/fact-sheets/detail/noncommunicable-diseases (accessed April 21, 2022).

[2] Katan M Luft A. Global burden of stroke. Semin Neurol. (2018) 38:208–11. 10.1055/s-0038-1649503 29791947

[3] Naghavi M Abajobir AA Abbafati C Abbas KM Abd-Allah F Abera SF. Global, regional, and national age-sex specific mortality for 264 causes of death, 1980–2016: a systematic analysis for the Global Burden of Disease Study 2016. Lancet. (2017) 390:1151–210. 10.1016/S0140-6736(17)32152-9 28919116 PMC5605883

[4] GBD 2016 Lifetime Risk of Stroke Collaborators. Global, regional, and country-specific lifetime risks of stroke, 1990 and 2016. N Engl J Med. (2018) 379:2429–37. 10.1056/NEJMoa1804492 30575491 PMC6247346

[5] Hay SI Abajobir AA Abate KH Abbafati C Abbas KM Abd-Allah F. Global, regional, and national disability-adjusted life-years (DALYs) for 333 diseases and injuries and healthy life expectancy (HALE) for 195 countries and territories, 1990–2016: a systematic analysis for the Global Burden of Disease Study 2016. Lancet. (2017) 390:1260–344. 10.1016/S0140-6736(17)32130-X 28919118 PMC5605707

[6] Vos T Abajobir AA Abate KH Abbafati C Abbas KM Abd-Allah F. Global, regional, and national incidence, prevalence, and years lived with disability for 328 diseases and injuries for 195 countries, 1990–2016: a systematic analysis for the Global Burden of Disease Study 2016. Lancet. (2017) 390:1211–59. 10.1016/S0140-6736(17)32154-2 28919117 PMC5605509

[7] Feigin VL Brainin M Norrving B Martins S Sacco RL Hacke W. World Stroke Organization (WSO): global stroke fact sheet 2022. Int J Stroke. (2022) 17:18–29. 10.1177/17474930211065917 34986727

[8] Abd-Allah F Khedr E Oraby M Reda R. Stroke burden in Egypt: data from five epidemiological studies. J Neurol Sci. (2018) 405:9–10. 10.1080/00207454.2017.1420068 29258372

[9] El-Hajj M Salameh P Rachidi S Hosseini H. The epidemiology of stroke in the Middle East. Eur Stroke J. (2016) 1:180–98. 10.1177/2396987316654338 31008279 PMC6453228

[10] Abd-Allah F Moustafa RR. Burden of stroke in Egypt: current status and opportunities. Int J Stroke. (2014) 9:1105–8. 10.1111/ijs.12313 25041503

[11] Bivard A Lin L Parsonsb MW. Review of stroke thrombolytics. J Stroke. (2013) 15:90. 10.5853/jos.2013.15.2.90 24324944 PMC3779670

[12] Reed M Kerndt CC Nicolas D. Alteplase. In: StatPearls. Treasure Island, FL: StatPearls Publishing (2023).

[13] Hacke W Kaste M Bluhmki E Brozman M Dávalos A Guidetti D. Thrombolysis with alteplase 3 to 4.5 hours after acute ischemic stroke. N Engl J Med. (2008) 359:1317–29. 10.1056/NEJMoa0804656 18815396

[14] Yamaguchi T Mori E Minematsu K Nakagawara J Hashi K Saito I. Alteplase at 0.6 mg/kg for acute ischemic stroke within 3 hours of onset. Stroke. (2006) 37:1810–5. 10.1161/01.STR.0000227191.01792.e3 16763187

[15] Lees KR Bluhmki E Von Kummer R Brott TG Toni D Grotta JC. Time to treatment with intravenous alteplase and outcome in stroke: an updated pooled analysis of ECASS, ATLANTIS, NINDS, and EPITHET trials. Lancet. (2010) 375:1695–703. 10.1016/S0140-6736(10)60491-6 20472172

[16] Patil S Rossi R Jabrah D Doyle K. Detection, diagnosis and treatment of acute ischemic stroke: current and future perspectives. Front Med Technol. (2022) 4:748949. 10.3389/fmedt.2022.748949 35813155 PMC9263220

[17] Multicenter Acute Stroke Trial–Europe Study Group Hommel M Cornu C Boutitie F Boissel JP. Thrombolytic therapy with streptokinase in acute ischemic stroke. N Engl J Med. (1996) 335:145–50. 10.1056/NEJM199607183350301 8657211

[18] Hacke W Kaste M Fieschi C Toni D Lesaffre E von Kummer R. Intravenous thrombolysis with recombinant tissue plasminogen activator for acute hemispheric stroke. The European Cooperative Acute Stroke Study (ECASS). JAMA. (1995) 274:1017–25. 10.1001/jama.274.13.1017 7563451

[19] Hacke W Kaste M Fieschi C von Kummer R Davalos A Meier D. Randomised double-blind placebo-controlled trial of thrombolytic therapy with intravenous alteplase in acute ischaemic stroke (ECASS II). Second European-Australasian Acute Stroke Study Investigators. Lancet. (1998) 352:1245–51. 10.1016/S0140-6736(98)08020-9 9788453

[20] World Bank Website. Death Rate Crude (per 1000). Available online at: https://data.worldbank.org/indicator/SP.DYN.CDRT.IN?end=2018&locations=EG&start=2018&view=bar (accessed January 7, 2020).

[21] Pan Y Chen Q Zhao X Liao X Wang C Du W. Cost-effectiveness of thrombolysis within 4.5 hours of acute ischemic stroke in China. PLoS ONE. (2014) 9:e110525. 10.1371/journal.pone.0110525 25329637 PMC4203798

[22] Emberson J Lees KR Lyden P Blackwell L Albers G Bluhmki E. Effect of treatment delay, age, and stroke severity on the effects of intravenous thrombolysis with alteplase for acute ischaemic stroke: a meta-analysis of individual patient data from randomized trials. Lancet. (2014) 384:1929–35. 10.1016/S0140-6736(14)60584-5 25106063 PMC4441266

[23] Tong X Wiltz JL George MG Odom EC Coleman King SM Chang T. A decade of improvement in door-to-needle time among acute ischemic stroke patients, 2008 to 2017. Circulation. (2018) 11:e004981. 10.1161/CIRCOUTCOMES.118.004981 30557047 PMC6329285

[24] Rajan SS Decker-Palmer M Wise J Dao T Salem C Savitz SI. Beneficial effects of the 30-minute door-to-needle time standard for alteplase administration. Ann Clin Transl Neurol. (2021) 8:1592–600. 10.1002/acn3.51400 34247448 PMC8351388

[25] Tatar M Sentürk A Tetik E Yildiz L Cheynel J. Budget impact of alteplase in treatment of acute ischemic stroke in Turkey. Value Health. (2017) 20:A607. 10.1016/j.jval.2017.08.1183

[26] Mar J Arrospide A Comas M. Budget impact analysis of thrombolysis for stroke in Spain: a discrete event simulation model. Value Health. (2010) 13:69–76. 10.1111/j.1524-4733.2009.00655.x 19818059

[27] Ehlers L Andersen G Clausen LB Bech M Kjølby M. Cost-effectiveness of intravenous thrombolysis with alteplase within a 3-hour window after acute ischemic stroke. Stroke. (2007) 38:85–9. 10.1161/01.STR.0000251790.19419.a8 17122430

[28] Boudreau DM Guzauskas GF Chen E Lalla D Tayama D Fagan SC. Cost-effectiveness of recombinant tissue-type plasminogen activator within 3 hours of acute ischemic stroke: current evidence. Stroke. (2014) 45:3032–9. 10.1161/STROKEAHA.114.005852 25190439

[29] Holmes M Davis S Simpson E. Alteplase for the treatment of acute ischaemic stroke: a NICE single technology appraisal; an evidence review group perspective. Pharmacoeconomics. (2015) 33:225–33. 10.1007/s40273-014-0233-z 25424495

[30] Joo H Wang G George MG. A literature review of cost-effectiveness of intravenous recombinant tissue plasminogen activator for treating acute ischaemic stroke. Stroke Vasc Neurol. (2017) 2:73–83. 10.1136/svn-2016-000063 28736623 PMC5516524

[31] Worldpopulationreview.com. Cairo Population 2022 (Demographics, Maps, Graphs). (2022). Available online at: https://worldpopulationreview.com/world-cities/cairo-population (accessed June 10, 2022).

